# Improved Baculovirus Vectors for Transduction and Gene Expression in Human Pancreatic Islet Cells

**DOI:** 10.3390/v10100574

**Published:** 2018-10-20

**Authors:** Leo P. Graves, Mine Aksular, Riyadh A. Alakeely, Daniel Ruiz Buck, Adam C. Chambers, Fernanda Murguia-Meca, Juan-Jose Plata-Muñoz, Stephen Hughes, Paul R. V. Johnson, Robert D. Possee, Linda A. King

**Affiliations:** 1 Department of Biological and Medical Sciences, Oxford Brookes University, Oxford OX3 0BP, UK; l.graves@oetltd.com (L.P.G.); p0086632@brookes.ac.uk (R.A.A.); r.possee@oetltd.com (R.D.P.); 2 Oxford Expression Technologies Ltd., Bioinnovation Hub, Gipsy Lane Campus, Oxford OX3 0BP, UK; m.aksular@oetltd.com (M.A.); d.ruizbuck@oetltd.com (D.R.B.); a.chambers@oetltd.com (A.C.C.); 3 Department of Biotechnology, College of Sciences, Baghdad University, Baghdad 10071, Iraq; 4 Centre for Molecular and Cell-Based Therapeutics SA de CV, Mexico City 15820, Mexico; pfmurguia@gmail.com (F.M.-M.); jj.platamunoz@gmail.com (J.-J.P.-M.); 5 Nuffield Department of Surgical Sciences, University of Oxford, Oxford OX3 9DU, UK; stephen.hughes@nds.ox.ac.uk (S.H.); paul.johnson@nds.ox.ac.uk (P.R.V.J.)

**Keywords:** BacMam, baculovirus, gene therapy, high-titre virus, human pancreatic islet cells

## Abstract

Pancreatic islet transplantation is a promising treatment for type 1 diabetes mellitus offering improved glycaemic control by restoring insulin production. Improved human pancreatic islet isolation has led to higher islet transplantation success. However, as many as 50% of islets are lost after transplantation due to immune responses and cellular injury, gene therapy presents a novel strategy to protect pancreatic islets for improved survival post-transplantation. To date, most of the vectors used in clinical trials and gene therapy studies have been derived from mammalian viruses such as adeno-associated or retrovirus. However, baculovirus BacMam vectors provide an attractive and safe alternative. Here, a novel BacMam was constructed containing a frameshift mutation within *fp25*, which results in virus stocks with higher infectious titres. This improved *in vitro* transduction when compared to control BacMams. Additionally, incorporating a truncated vesicular stomatitis virus G protein increased transduction efficacy and production of EGFP and BCL2 in human kidney (HK-2) and pancreatic islet β cells (EndoC βH3). Lastly, we have shown that our optimized BacMam vector can deliver and express *egfp* in intact pancreatic islet cells from human cadaveric donors. These results confirm that BacMam vectors are a viable choice for providing delivery of transgenes to pancreatic islet cells.

## 1. Introduction

Islets of Langerhans are micro-organs that comprise a cluster of cells consisting of glucagon-secreting alpha cells, insulin-secreting beta cells, pancreatic polypeptide-secreting F (or gamma) cells, somatostatin-secreting delta cells, and ghrelin-secreting epsilon cells [1,2]. The pancreas contains between 300,000 and 1.5 million islets, which contribute 1–2% of the total pancreatic mass [3].

Type 1 diabetes mellitus (DM1), which has been diagnosed in an estimated 35 million patients worldwide, is caused by auto-immune destruction of the pancreatic islet β cells and subsequent insulin deficiency [3,4]. Although DM1 is less common than other type of diabetes, it remains a serious chronic disorder usually starting during childhood or adolescence [5]. Whilst in most people the disease can be managed through daily injection of insulin, some suffer acute hypoglycaemic episodes that may be life-threatening [6]. For these patients, one of the most promising therapies is pancreatic islet transplantation as it is a minimally invasive treatment that has the potential to reverse DM1. This leads to improved glycaemic control, abrogating the need for insulin in some patients [7,8].

Most islet recipients require more than one infusion to achieve insulin-independence as both pancreatic islet isolation and survival after transplantation determine the success of the procedure [9]. Although some loss in yield occurs during pancreatic islet cell isolation, several studies have shown that most of the losses occur due to pancreatic islet death in the post-transplant period [10,11].

Many cellular mechanisms contribute to pancreatic islet destruction following transplantation including alloantigen-specific and immune-mediated destruction. However, most losses have been attributed to inflammatory events caused mainly by ischemia-reperfusion injury (IRI) [12,13,14]. Damage from IRI occurs when the blood supply returns to the tissue after a period of anoxia and nutrient depletion [15]. Molecular oxygen, present in the restored blood supply, is the source of the reactive oxygen species (ROS) responsible for inflammation and oxidative damage in transplanted tissue [16]. Previous studies have shown that apoptosis caused by IRI is responsible for the induction of inflammation and subsequent organ damage. Moreover, it has been shown that suppression of apoptosis can prevent inflammation and tissue injury [17,18].

It is possible that pre-transplantation gene therapy could be used to improve the outcomes of pancreatic islet transplantation. Gene therapy involves the therapeutic delivery of specific gene(s) into target cells and could be used as a tool to improve islet transplant success by delivering genes that could reduce IRI, inflammation or inhibit apoptosis. Viruses are good candidates to be used as gene therapy vectors as they have evolved to enter host cells and deliver genetic material for gene expression. The majority of work in this field has utilised mammalian viruses such as adeno-associated, retro and herpes viruses. However, there are safety concerns with using mammalian viruses due to their natural pathogenicity and immunogenicity [19]. An alternative insect-specific virus vector based on baculovirus does not have any of these safety concerns. In addition, there is no pre-existing immunity to baculovirus unlike mammalian virus-based vectors [20].

Baculoviruses are arthropod-specific viruses widely used for high-yield protein production in insect cells with most vectors based on the Autographa californica nucleopolyhedrovirus (AcMNPV) [21]. These viruses are unable to replicate in mammalian cells as the viral promoters are not active. However, the baculovirus can transduce a wide variety of mammalian cells and express foreign genes when these are placed under the control of a mammalian promoter such as the cytomegalovirus (CMV) immediate early 1 promoter. Baculovirus-based vectors for expression in mammalian cells are referred to as BacMam [22,23,24,25].

The main disadvantage of the BacMam system is that relatively high ‘multiplicities of infection’ (100+ virus particles per cell) are necessary to deliver sufficient virus genomes into target cells for effective transgene expression [26,27]. The input virus does not replicate so gene expression depends solely on the number of genomes that enter the cell. Therefore, the BacMam virus can require either concentration prior to the transduction or the use of chemicals to enhance gene expression, e.g., sodium butyrate [28]. Concentration of virus is possible but time-consuming and labour intensive. The use of chemical enhancers may have side effects on cell metabolism, which will be undesirable if the intended use of the BacMam is gene therapy.

Here, we report the construction of a novel BacMam virus that contains a mutation within *fp25*, which comprises the insertion of an additional adenine that causes a frame-shift in the coding region, truncating the native FP25 protein [29]. When using baculovirus vectors in insect cells, this mutation is undesirable as it reduces expression from the polyhedrin gene promoter due to a decrease in the rate of transcription [30]. Conversely, however, this mutation has also been shown to enable production of virus stocks with consistently very high infectious titres [29,31]. In this study, we evaluate the benefits of incorporating the *fp25* ‘high-titre’ (HT) mutation into a BacMam genome for the transduction of mammalian cells.

The molecular mechanisms involved in BacMam entry into mammalian cells remain poorly characterized. However, despite this, some studies have demonstrated that BacMam transduction efficacy can be significantly improved by displaying different proteins on the baculovirus budded virus (BV) surface [32,33]. In the current study, we combined the *fp25* HT mutation genome with pseudotyping the baculovirus envelope with a truncated vesicular stomatitis virus-G (VSV-G) protein. The benefits of this new vector for mammalian cell transduction and gene expression was evaluated in cell culture and in human pancreatic islet cells.

## 2. Materials and Methods

### 2.1. Cells, Plasmids and Viruses

#### 2.1.1. Cells

Insect cell lines *Spodoptera frugiperda* Sf9 [34] and Sf21 [35] were maintained at 28 °C using ESF921 media (Expression Systems) or TC100 media supplemented with 10% (*v*/*v*) foetal calf serum (Thermo Fisher Scientific, Loughborough, UK; TFS), respectively. HK-2 cells were purchased from American Type Culture Collection and cultured in Keratinocyte serum-free medium (KSFM; GLT) supplemented with human recombinant Epidermal Growth Factor 1-53 (EGF 1-53) and Bovine Pituitary Extract (BPE). Cells were incubated at 37 °C with 5% (*v*/*v*) CO_2_. The EndoC-βH3^®^ cell line was purchased from Univercell-Biosolutions, Toulouse, France (U-B) and cultured according to the user’s guide in OPTlβ1^®^ media (U-B) containing 5 µg/mL puromycin [36]. Cells were seeded onto βCOAT^®^-treated TPP^®^ tissue culture flasks (U-B) at 7 × 10^4^ cells/cm^2^.

#### 2.1.2. Transfer Plasmid Construction

To generate the pCMV.EGFP plasmid, *egfp* was excised from pEGFP-N1 (Clontech, Mountain View, CA, USA) with restriction endonucleases *Not*I and *Eco*RI and inserted into pOET6-BacMam, which contains the CMV immediate-early 1 enhancer promoter (Oxford Expression Technologies, Oxford, UK; OET). For pCMV.BCL2, *bcl2* was PCR amplified from a synthetic gene (GeneArt™) to introduce *Eco*RI and *Xba*I sites at the 5′ and 3′ ends, respectively, and also inserted into pOET6-BacMam. pAcCMV.EGFP_VSV-G was generated in several steps. The pEGFP-N1 was modified by inserting an *Xba*I linker into the unique *Ase*I site. The CMV promoter-EGFP gene cassette was then removed from this vector with *Xba*I and *Not*I and inserted into pAcUW21 [37] previously modified to include a *Not*I site between unique *Xba*I and *Bgl*II sites. A 678 bp synthetic sequence comprising the AcMNPV polyhedrin gene (*polh*) promoter and *gp64* signal peptide coding region linked with the truncated version of VSV-G [32] was then inserted between the *Xba*I and *Swa*I sites of this vector to create pAcCMV.EGFP_VSV-G.

#### 2.1.3. BacMam Production

Recombinant BacMam were prepared by mixing transfer plasmids with either *flash*BAC™ or BacPAK6^HT^ virus DNA and transfecting Sf9 cells as recommended by the supplier (OET). After 5 days of incubation at 28 °C, the cell media containing budded viruses were harvested and stored at 4 °C. BacMams for generating high titre viruses were prepared by first constructing BacPAK6^HT^. This was achieved by co-transfecting AcdefrTp35r [29] with pBacPAK6 and selecting a recombinant virus with a copy of the β-galactosidase coding region under the control of the *polh* promoter essentially as described previously [38]. Virus DNA was extracted from BacPAK6^HT^, digested with *Bsu*36I and mixed with transfer vectors prior to co-transfecting Sf9 cells. Recombinant BacMam were selected and plaque purified to genetic homogeneity [38].

#### 2.1.4. Virus Amplification

Additional passages of the virus stocks were amplified in Sf9 cells as previously described [39]. Sf9 cells were seeded at a density of 1.5–2 × 10^6^ cells/mL in a shake culture, and then infected with the virus at a multiplicity of infection (MOI) of 0.1 pfu/cell and incubated for 5 days at 28 °C on a shaking platform. After incubation, the culture was harvested and centrifuged at 4000 rpm for 15 min at 4 °C (TY-JS 4.2 rotor, J6-MI Beckman centrifuge) to remove cell debris. The clarified culture medium containing BV was stored in aliquots at 4 °C.

#### 2.1.5. Titration of Virus Infectivity

Baculoviruses stocks were titrated either using a conventional method based on a plaque assay [39] or more rapidly with a quantitative polymerase chain reaction technique (*baculo*QUANT™) as described by the manufacturer (OET).

### 2.2. Gene Delivery in Mammalian Cells Using BacMam Virus Vectors

#### 2.2.1. In Vitro Gene Delivery

One mL HK-2 cells were seeded at a concentration of 1 × 10^5^ cells/mL in a 12-well plate and incubated overnight at 37 °C. The cells were then washed with sterile phosphate buffered saline (PBS) and transduced with BacMam viruses at a MOI of 150 pfu/cell and incubated for 5 h at 37 °C. The virus inoculum was then replaced with growth medium and the cells were returned to 37 °C for the required time. EndoC-βH3 cells were seeded onto βCOAT^®^ (U-B) treated 12-well plates at 2.5 × 10^5^ cells/well. The following day, cells were washed with sterile PBS and then transduced with BacMam viruses using a MOI of 250 pfu/cell and incubated for 5 h at 37 °C. The virus inoculum was then replaced with growth medium and cells were returned to 37 °C for the required time. Inducible excision of CRE-mediated immortalizing transgenes was performed with addition of 4-Hydroxy Tamoxifen (1 µM) for 3 weeks prior to transductions.

#### 2.2.2. Ex Vivo Donor Islet Cells

Islet cells were supplied by the Diabetes Research and Wellness Foundation (DRWF) human islet isolation facility, Churchill Hospital, Oxford in cold storage media. On arrival, an estimation of the total cell numbers was calculated from a small aliquot. The islets were then re-suspended in fresh medium (CRML media (Gibco™) containing l-Glutamine (1×) and 2% human albumin), seeded at a concentration of 1 × 10^5^ cells/well in a 12-well plate and incubated for 1 h at 37 °C with 5% (*v*/*v*) CO_2_ to recover. The islets were then directly transduced using a MOI of 150 pfu/cell and the incubation continued for up to 48 h after transduction.

### 2.3. Fluorescence Microscopy

Fluorescence microscopy was performed on *in vitro* and *ex vivo* samples at different time points using a Zeiss Axiovert 135 inverted epifluorescence microscope (Cambridge, UK) with a 10× Plan Neofluar objective lens and 10× ocular lens. For EGFP detection, a band pass 546 filter was used.

### 2.4. Fractionation of Budded Virus Envelope

Separation of purified BV into envelope and capsid fractions was performed essentially as previously described [40]. Briefly, purified BV particles were re-suspended in 1% (*v*/*v*) NP-40 for 30 min and then the capsid fraction was separated by centrifugation.

### 2.5. SDS-PAGE and Immunoblot Analysis

Proteins were separated on 4–20% mini-PROTEAN^®^TGX™ pre-cast protein gels (Bio-Rad, Watford, UK; B-R) and transferred onto a polyvinylidene diflouride (PVDF) membrane using a Trans-Blot^®^Turbo™ transfer pack (B-R). Following the transfer, membranes were incubated in 5% powdered milk (Marvel^®^) dissolved in PBST (1× PBS containing 0.1% (*v*/*v*) Tween20) and agitated at room temperature for 1 h to prevent non-specific binding. Subsequently, membranes were treated with either rabbit polyclonal EGFP, mouse monoclonal actin (Abcam, Cambridge, UK), mouse monoclonal BCL2 (Santa Cruz, Dallas, TX, USA) or rabbit monoclonal VSV-G (Abcam) primary antibodies for 1 h. Membranes were washed 4 × 10 min with PBST and incubated with a species-specific secondary antibody conjugated with horseradish peroxidase for 1 h. To remove any background, membranes were washed 4 × 10 min with PBST. Bound proteins were visualized using Clarity™ Western ECL blotting substrates (B-R). After a 5 min incubation, the bands were imaged using a ChemiDoc™MP imaging system (B-R). The band intensities for actin (a^i^) were used as an internal reference for each sample to calculate the amount of synthesis of either protein targets (t^i^) EGFP or BCL2 (a^i^/t^i^ × 100), expressed as a percentage increase.

### 2.6. Statistical Analysis

All data were analysed using GraphPad Prism Version 7 for Windows (GraphPad Software Inc., La Jolla, CA, USA) with results displayed in ± SD. Statistical analysis were performed using t-test, with a *p*-value of < 0.05 considered statistically significant.

### 2.7. Flow Cytometry

Flow cytometry was used to provide a quantitative measure of percentage transduced cells and the intensity of the signal within cells. Green fluorescence from *in vitro* and *ex vivo* BacMam-transduced cells, harvested at different time points, were analysed using a Novocyte 3000 Flow Cytometer (ACEA Biosciences, San Diego, CA, USA) according to the manufacturer’s instructions. Negative gates were set using the data from mock-transduced cells.

### 2.8. Confocal Microscopy

BacMam-transduced islet cells were washed twice in PBS before fixation for 45 min at room temperature with 4% formaldehyde in PBS. The fixative was removed and islets were washed twice in PBS prior to being re-suspended in Vectashield mounting medium with DAPI (Vector Laboratories, Peterborough, UK) onto glass slides. The fixed islets were covered with glass cover slips and stored at 4 °C until imaging. Images were acquired using an oil immersion objective (Plan-Apochromat 63X, 1.4 numerical aperture) attached to a Zeiss LSM 880 laser scanning microscope. Post-acquisition image processing and Z-stack image projections were processed using ZEN black software (Zeiss, Cambridge, UK).

## 3. Results

### 3.1. Enhancing Infectious Budded Virus Production Using BacMam with a Mutation in fp25

**Figure 1:**
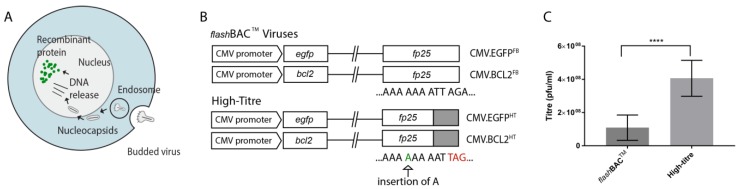
(**A**) schematic representation of BacMam mediated gene delivery into mammalian cells (transduction). Budded viruses are taken up by endocytosis and released into the cytoplasm. The nucleocapsids are directed to the nucleus where the DNA is released for transcription under the control of a mammalian promoter and the resultant mRNA is then translated into the recombinant protein; (**B**) schematic representation of *flash*BAC™ (FB) and high-titre (HT) *egfp* and *bcl2* BacMam vectors. The *fp25* mutation results from the insertion of an adenine causing a frameshift and an early stop codon (red letters); (**C**) comparison of the infectious titres from 10 FB and HT BacMam viruses as determined by plaque assay. Results were plotted using Graphpad Prism (error bars represent ± SD) and analysed using a Student’s *t*-test (*p* < 0.05).

Baculoviruses are able to enter mammalian cells and express foreign genes placed under the control of a mammalian gene promoter in a process known as transduction (Figure 1A). To explore the feasibility of using these vectors for ex vivo gene therapy of pancreatic islet cells, BacMam viruses expressing enhanced green fluorescent protein (*egfp*) under the control of the CMV immediate early 1 promoter were generated to observe and monitor transduction efficacy. An additional virus vector encoding an anti-apoptotic gene B-cell lymphoma-2 (*bcl2*) was constructed to investigate the expression of a therapeutic gene using the BacMam system (Figure 1B).

The BacMam vectors generated in this study were based on two parental virus genomes. The first comprised *flash*BAC™ (FB), normally used to make recombinant viruses for expression of genes in insect cells. The second contained a single nucleotide insertion in *fp25*, which results in a frame-shift mutation that introduces an early stop codon into the coding region of the gene (Figure 1B). This mutation has been shown to decrease polyhedra formation and increase BV production [30,41]. Hence, this virus was designated high-titre (HT). To explore the effect of this mutation on BV production by recombinant BacMam vectors, ten different FB and HT viruses were prepared and their infectivity determined. The average titres for the FB and HT viruses were 1.09 × 10^8^ and 4.07 × 10^8^, respectively. This four-fold increase in the average infectious titre for the HT viruses was shown to be statistically significant (Figure 1C).

### 3.2. Improving BacMam-Mediated Gene Expression in Mammalian Cells

**Figure 2:**
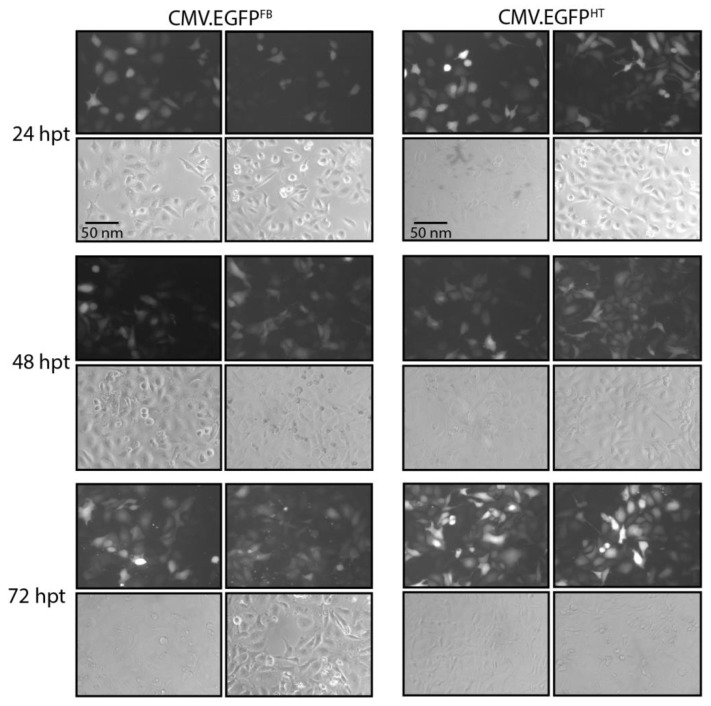
Representative images of bright (lower panels) and fluorescent (upper panels) fields of HK-2 cells transduced with CMV.EGFP^FB^ (FB) or CMV.EGFP^HT^ (HT) BacMam viruses at ‘multiplicity of infection’ of 150. Images were taken 24, 48 and 72 hpt using a Zeiss Axiovert 135 inverted epifluorescence microscope (10×). Duplicate images are shown for each virus and time point. Scale-bar, 50 nm.

In order to compare transduction efficacy between the FB and HT BacMam vectors, expression of *egfp* and *bcl2* was first evaluated in human kidney (HK-2) cells using CMV.EGFP^FB^, CMV.EGFP^HT^, CMV.BCL2^FB^ or CMV.BCL2^HT^. A null virus (CMV.NULL^HT^), lacking a gene under the CMV immediate early gene promoter, and mock-transduced cells, were included as negative controls in all experiments. Transductions were carried out in triplicate and recombinant protein production was evaluated by fluorescent microscopy, flow cytometry and Western blotting using target-specific antibodies. Initial comparisons between CMV.EGFP^FB^- and CMV.EGFP^HT^-transduced HK-2 cells using fluorescence microscopy showed that *egfp* expression was detected at 24 h post-transduction (hpt) and continued to increase up to 72 hpt (Figure 2). A greater number of cells, and a higher intensity of fluorescence within cells, was observed in transductions with CMV.EGFP^HT^ compared with CMV.EGFP^FB^ (Figure 2).

To provide a quantitative measure of transduction efficacy, CMV.EGFP^FB^- or CMV.EGFP^HT^-transduced HK-2 cells were analysed using flow cytometry. Interestingly, these results (Table 1) demonstrated that by 24 hpt, a high percentage of cells had been successfully transduced by both CMV.EGFP^FB^ (90% ± 1.6) and CMV.EGFP^HT^ (95% ± 0.97). By 48 hpt, almost all cells (99%) showed evidence of successful transduction with either the FB or HT vector. However, the intensity of EGFP fluorescence in CMV.EGFP^HT^-transduced HK-2 cells was higher than those transduced with CMV.EGFP^FB^, as shown by a higher proportion of cells with a fluorescein isothiocyanate (FITC) reading above 10^7^ (Table 1) at all times points after transduction. Overall, these results suggest that BacMam vectors incorporating the HT mutation do not enhance the proportion of cells transduced, but, instead, may help deliver more copies of the target gene per cell, which results in increased recombinant protein production (in this case, EGFP).

**Figure 3:**
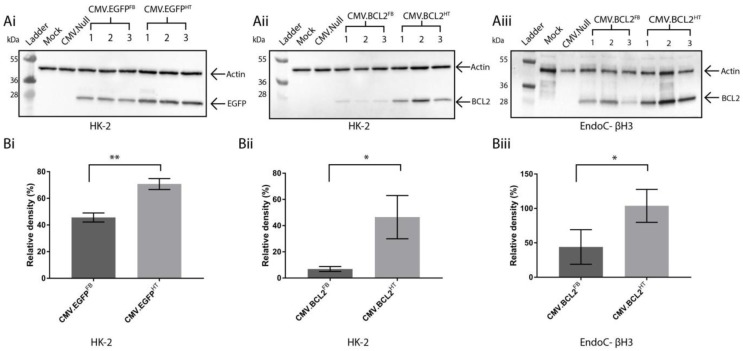
(**A**) immunoblot analysis of cell lysates following transduction with the named BacMam vector in HK-2 (**Ai**,**Aii**) or EndoC-βH3 (**Aiii**) cells at 72 hpt using anti-EGFP or anti-BCL2 antibodies. Molecular weight markers are in kDa. (**B**) A bar graph was constructed using GraphPad Prism to show the relative band intensities for (**Bi**) EGFP in HK-2, (**Bii**) BCL2 in HK-2 and (**Biii**) BCL2 in EndoC-βH3 cells obtained by band densitometry. Each sample was normalised against a β-actin loading control. Error bars represent ± SD (*n* = 3) and indicate a significant difference between CMV.EGFP^FB^ and CMV.EGFP^HT^, or CMV.BCL2^FB^ and CMV.BCL2^HT^ transduced cells using a Student’s *t*-test (*p* < 0.05).

To investigate any difference in recombinant protein production between the normal and HT BacMam vector, immunoblot analysis was performed on transduced HK-2 cell lysates harvested at 72 hpt (Figure 3A(i,ii)). This analysis demonstrated that the production of both EGFP and BCL2 was increased in HK-2 cells transduced with the HT BacMam vector in comparison to those transduced with the unmodified vectors. To semi-quantify these results, band densitometry was performed using a β-actin loading control for standardisation (Figure 3B(i,ii)). This demonstrated a statistically significant increase in protein production of approximately 25% (EGFP; Figure 3Bi) and 50% (BCL-2; Figure 3Bii) when HK-2 cells were transduced with CMV.EGFP^HT^ and CMV.BCL2^HT^, respectively.

To confirm whether the HT BacMam was the more suitable vector for gene delivery, it was pertinent to replicate the experiments in other cell lines. For the interests of this study, a human derived pancreatic beta cell line (EndoC-βH3) was selected, which would also provide an early indication of donor islet cell susceptibility to BacMam vectors. In addition, it was crucial to test whether the therapeutic gene target *bcl2* could be expressed in a pancreatic beta cell line. Therefore, EndoC-βH3 cells were transduced with CMV.BCL2^FB^ or CMV.BCL2^HT^ and harvested at 72 hpt (Figure 3Aiii). Western blot analysis confirmed the production of BCL2 and band densitometry analysis demonstrated an approximately 50% increase in protein yield using the HT vector in comparison to FB (Figure 3Biii). This provides further evidence that a BacMam vector based on the HT virus genome may be the more suitable choice to achieve improved gene delivery.

### 3.3. Pseudotyping Virus Particles with Truncated Vesicular Stomatitis Virus G-Protein to Enhance Transduction Efficacy

**Figure 4:**
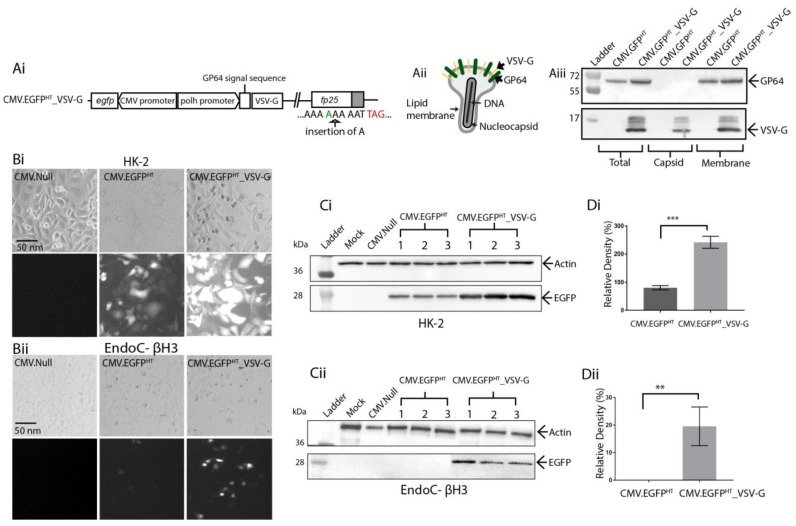
(**A**) schematic representation of the VSV-G pseudotyped BacMam vector construct. (**Ai**) 21-amino-acid ectodomain of VSV-G, with its transmembrane and cytoplasmic tail domains (aa 442–511) [29] was inserted into the HT virus genome under the control of the *polh* promoter as a fusion to the *gp64* signal peptide. TAG, stop codon (red) (**Aii**) Schematic representation of the BV particle with VSV-G incorporation in the envelope surface alongside the native GP64 protein. (**Aiii**) immunoblot analysis of intact and fractionated BV (nucleocapsid and membrane envelope) were analysed by Western blotting using anti-VSV-G or anti-GP64 specific antibodies. Molecular weight markers are in kDa. (**B**) bright field and fluorescence images of (**Bi**) HK-2 (MOI 150) and (**Bii**) EndoC-βH3 (MOI 250) cells transduced with CMV.EGFP^HT^ VSV-G pseudotyped or non-pseudotyped BacMam vectors. Images were taken at 72 hpt at 10× using a Zeiss Axiovert 135 inverted epifluorescence microscope. Scale bar, 50 nm. (**C**) immunoblot analysis of transduced (**Ci**) HK-2 and (**Cii**) EndoC-βH3 cell lysates harvested at 72 hpt, using target-specific antibodies. Β-actin was used as a loading control. Molecular weight markers in kDa; (**D**) a bar graph was constructed using GraphPad Prism showing the relative band intensities for EGFP in (**Di**) HK-2 and (**Dii**) EndoC-βH3 cells. Each sample was normalised against β-actin. Error bars represent ± SD (n = 3) and indicate a significant difference between CMV.EGFP^HT^ and CMV.EGFP^HT^_VSV-G transduced cells as analysed using a Student’s *t*-test (*p* < 0.05).

It has been shown that pseudotyping of viral vectors can be employed to enhance transduction of particles into cells [42,43]. Incorporation of a truncated VSV-G into the baculovirus BV membrane has been shown to increase transduction efficacy up to 15-fold *in vivo* and *in vitro* [32]. To demonstrate whether VSV-G pseudotyping might have a similar effect on BacMam based on the HT genome, an additional vector was generated by inserting the truncated VSV-G gene under control of the *polh* promoter (Figure 4A(i,ii)). Incorporation of VSV-G into the BV envelope was confirmed by separating purified BV into nucleocapsid and envelope fractions, followed by immunoblot analysis using anti-VSV-G specific antibody (Figure 4Aiii).

The potential benefit of VSV-G pseudotyping was tested by transducing HK-2 (150 MOI) and EndoC-βH3 (250 MOI) cells with CMV.EGFP^HT^ or CMV.EGFP^HT^_VSV-G viruses (Figure 4B(i,ii)). Transduced cells were analysed at 72 hpt using fluorescence microscopy and Western blotting. Fluorescence microscopy showed that CMV.EGFP^HT^_VSV-G virus both increased the percentage of cells transduced and the intensity of EGFP fluorescence, when compared to the non-pseudotyped virus, in both cell lines tested (Figure 4B(i,ii)). This difference was more evident for EndoC-βH3 cells, where a very low level of *egfp* expression was detected when the cells were transduced with CMV.EGFP^HT^. In contrast, the pseudotyped vector resulted in considerably higher *egfp* expression. No *egfp* expression was detected in mock- and CMV.NULL^HT^-transduced cells (Figure 4B(i,ii)).

Immunoblot analysis supported the results observed with fluorescence microscopy; an increase in protein yield was detected from the VSV-G-pseudotyped vector-transduced cells (Figure 4C(i,ii)). As described earlier, this was further confirmed by band densitometry where EGFP synthesis by different recombinant viruses was assessed in comparison to an internal actin control. There was an increase in EGFP production of approximately 160% in HK-2 cells (Figure 4Di) and 20% in EndoC-βH3 cells (Figure 4Dii).

### 3.4. In Vitro Expression of egfp in a Non-Proliferative Human Beta Cell Line

**Figure 5:**
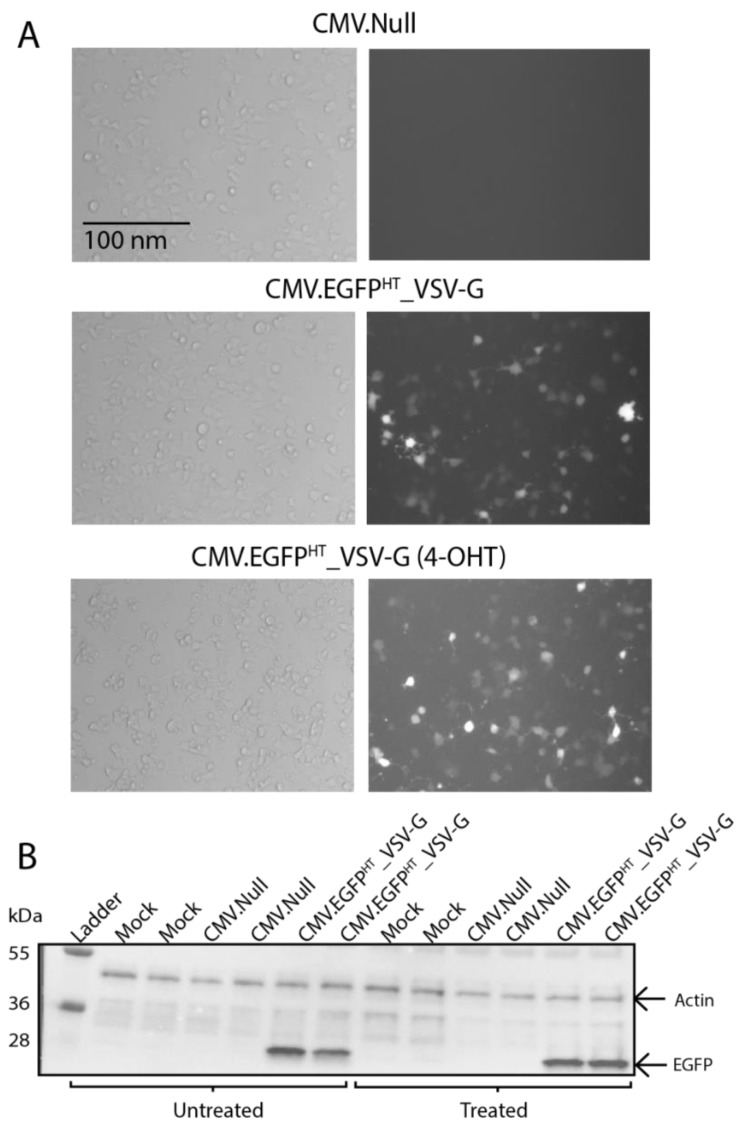
(**A**) bright field (left) and fluorescence (right) images of tamoxifen-treated (4-OHT) and untreated EndoC-βH3 cells transduced with CMV.EGFP^HT^_VSV-G at MOI 250. Treated cells were incubated with tamoxifen for three weeks prior to transduction to remove the CRE-mediated immortalising transgenes. Images were taken at 48 hpt using a Zeiss Axiovert 135 inverted epifluorescence microscope (10×). Scale bar, 100 nm. (**B**) Tamoxifen-treated or untreated EndoC-βH3 cells were transduced with the indicated BacMam vectors or controls (in duplicate) and were harvested at 48 hpt for analysis by Western blotting using EGFP-specific antibody. β-actin was used as a loading control. Molecular weight markers are in kDa.

The EndoC-βH3 cells were treated with tamoxifen to remove the CRE-mediated immortalising transgenes. After treatment, the cells become functional non-proliferative human β cells that closely represent the characteristics of pancreatic β cells and thus represent a more realistic model for pancreatic islets [36]. Due to the scarcity of donor islets, this provided an alternative model for studying the BacMam system in relation to successful gene delivery in pancreatic β cells. Both tamoxifen-treated and non-treated EndoC-βH3 cells were transduced, in duplicate, with the BacMam virus CMV.EGFP^HT^_VSV-G (Figure 5). Fluorescence microscopy and Western blot analyses of *egfp* expression at 48 hpt demonstrated that there was no decrease in the transduction susceptibility of the non-proliferating β cells to CMV.EGFP^HT^_VSV-G (Figure 5A,B).

### 3.5. BacMam Mediated Gene Expression in Human Pancreatic Islet Cells from Cadaveric Donors

**Figure 6:**
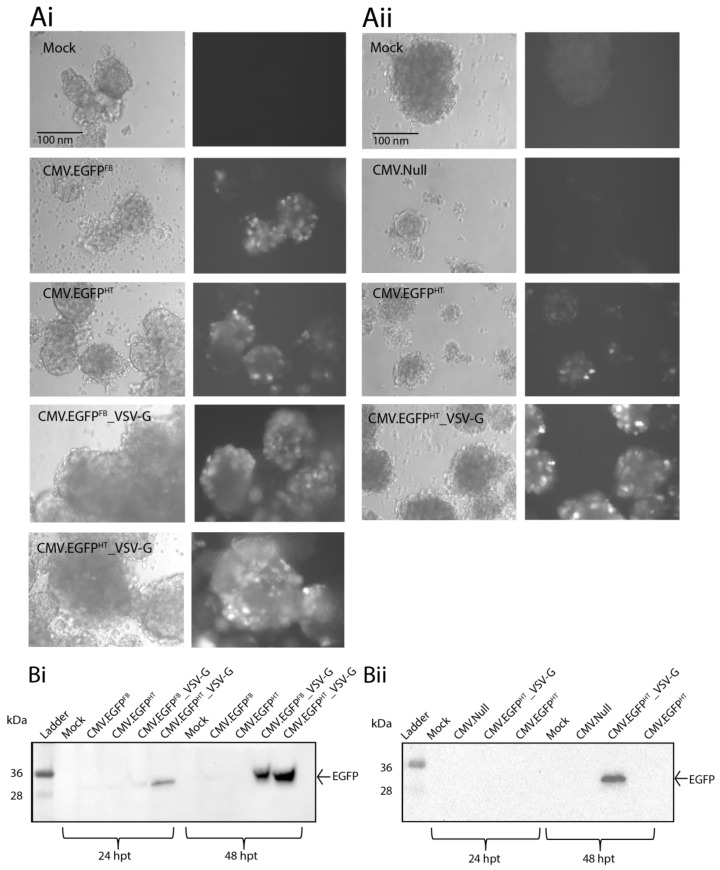
(**A**) human pancreatic islets isolated from cadaveric donor 1 (**Ai**) and donor 2 (**Aii**) were transduced with BacMam viruses at 250 MOI as shown. Bright field (left) and fluorescent (right) images were taken at 48 hpt using a Zeiss Axiovert 135 inverted epifluorescence microscope (×10). Scale bar, 100 nm. (**B**) Cell lysates were harvested from donor 1 (**Bi**) and donor 2 (**Bii**) at 24 and 48 hpt and were analysed by Western blotting using EGFP-specific antibodies. Molecular weight markers are in kDa.

Islets from two different cadaveric donors were transduced *ex vivo* with four different *egfp*-expressing BacMam vectors (CMV.EGFP^FB^, CMV.EGFP^HT^, CMV.EGFP^FB^_VSV-G and CMV-EGFP^HT^_VSV-G) using MOI 250 for each (Figure 6). The islets were monitored over 48 h and visual examination after BacMam-transduction did not appear to impact cell viability or cause the islet clusters to break apart. Fluorescence microscopy analysis demonstrated that *egfp* expression could be detected in transduced cells from all four vector versions at 48 hpt (Figure 6A). Partial auto-fluorescence was observed in both mock- and CMV.NULL^HT^-transduced samples (Figure 6A), however, this was likely a result of poor cell quality and/or cell stress, and was negligible in comparison to fluorescence associated with BacMam EGFP virus vectors (Figure 6A).

The difference in *egfp* expression levels observed between the standard FB and HT virus vectors was minimal. However, a marked increase was observed when either of the VSV-G-pseudotyped vectors (CMV.EGFP^FB^_VSV-G or CMV.EGFP^HT^_VSV-G) was used (Figure 6A). These initial observations from the two sets of islets suggested that pseudotyping the baculovirus BV with VSV-G results in improved gene delivery. To obtain a quantitative measure of this suggested improvement, CMV.EGFP^HT^- or CMV.EGFP^HT^_VSV-G-transduced islet cells from the second cadaveric donor were analysed by flow cytometry. The results indicated that at 48 hpt, 23.87% of the CMV.EGFP^HT^-transduced cells contained detectable EGFP compared to 34.64% of the CMV.EGFP^HT^_VSV-G-transduced cells.

In addition to the fluorescence microscopy and flow cytometry analyses, immunoblotting was carried out on transduced islets harvested at 24 and 48 hpt to assess the level of EGFP production. A stained gel was also performed on samples to ensure equal loading (data not shown). These results confirmed that the highest EGFP yield was obtained with CMV.EGFP^HT^_VSV-G at 48 hpt (Figure 6B). In addition, the lower EGFP yield obtained with CMV.EGFP^FB^_VSV-G compared with CMV.EGFP^HT^_VSV-G pseudotyped virus strengthens a role for the HT virus genome in improving transduction outcomes (Figure 6B). The absence of EGFP by immunoblotting for CMV.EGFP^FB^ and CMV.EGFP^HT^ is likely due to levels of EGFP being too low for detection.

**Figure 7:**
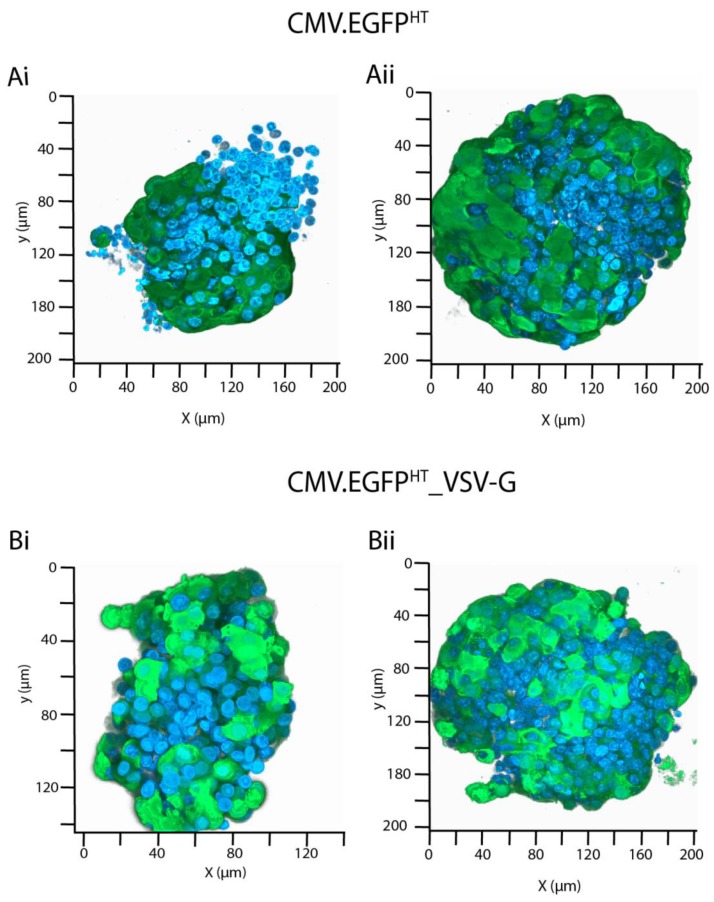
Human pancreatic islets isolated from cadaveric donor 2 were transduced with (**A**) CMV.EGFP^HT^ or (**B**) CMV.EGFP^HT^_VSV-G BacMam viruses at 250 MOI (two representative images are presented for each). Confocal microscopy images were taken at 48 hpt using a Zeiss LSM 880 laser scanning microscope (×63) and Z-stack image projections were processed using ZEN black (Zeiss, Cambridge, UK). Green = EGFP, Blue = DAPI.

As the islets comprised clusters of several hundred cells, and the flow cytometry analysis had indicated that only about one-third of cells, maximally, were transduced with BacMam, we were interested in understanding further the distribution of transduced cells within the islet cluster. Therefore, three-dimensional analysis of CMV.EGFP^FB^ or CMV.EGFP^FB^_VSV-G transduced islets was undertaken using confocal microscopy (z-stack function). The results (Figure 7) indicated considerable variation in the numbers of *egfp* expressing cells per islet for both CMV.EGFP^HT^ (Figure 7A(i,ii)) and CMV.EGFP^HT^_VSV-G (Figure 7B(i,ii)) at 48 hpt. This disparity is most likely related to the BacMam viruses being unable to infiltrate the inner cells of the larger islets.

## 4. Discussion

Pancreatic islet transplantation is being increasingly used as a treatment option for patients with severe DM1. This treatment uses purified donor islet cells that are transplanted into the hepatic portal circulation to promote engraftment. After surgery, the islet cells have been shown to offer improved glycaemic control through restoration of insulin production [44]. However, islet cell transplantation is limited by post-transplantation factors that can impact islet survival and longevity. These include graft rejection, blood-mediated inflammatory reaction and hypoxia [13]. Viruses have been adapted to efficiently deliver therapeutic genes [45] and to modify islets for improved survival post-transplantation [44,46]. The development of mammalian viral vectors capable of transferring therapeutic genes has already been used for a variety of metabolic disorders and autoimmune diseases [47], which account for 70% of clinical trials and gene therapy studies [40]. For islet transplantation, adeno-associated virus (AAV) has been evaluated due to its safety profile, broad cell tropism and accessibility of established helper cell lines for vector production [44,45,48,49]. However, AAV vectors are limited by a maximum size gene insert of <4.5 kb and scale-up issues resulting from difficulties in generating high-titres of virus [45,48]. Furthermore, mammalian virus vectors still pose several problems regarding pre-existing immunity in the patient and the resulting toxicity caused by adverse immune responses [50]. Therefore, this study investigated the use of baculoviruses, which are non-pathogenic and replication-incompetent in humans, to overcome the obstacles posed by currently used viral vectors [28,50,51,52]. In addition, this study demonstrates the development of a new BacMam vector that could be used to efficiently transfer therapeutic genes into human pancreatic islet cells.

The BacMam system requires relatively high MOI for effective transduction of mammalian cells. Therefore, to overcome this shortcoming, a BacMam vector that contains a mutation within *fp25*, which results in the production of infectious BV titres consistently above 3.0 × 10^8^ pfu/mL was developed. A BacMam vector with increased BV titre (HT BacMam) reduces the total volume of virus inoculum to be scaled-up and minimises the need for virus concentration protocols to reach the high MOI required for transduction. This could have future benefits resulting from reduced production and scale-up costs. The increased virus titres observed in this study were similar to previous findings, which indicates that the placement of a foreign gene under the CMV promoter did not impact the *fp25* mutant phenotype [30]. This phenotype has been attributed to a failure to shift from BV production to occlusion derived virus during the later phases of baculovirus infection, resulting in the continued production of BV [30,41].

The initial *in vitro* transduction experiments using two distinct cell lines, HK-2 and EndoC-βH3, demonstrated an increase in gene delivery for the HT BacMam viruses when compared to the standard (FB) BacMam viruses. Given the cells were transduced with equal MOI for both HT and FB viruses, the increased expression levels are likely to relate to improved BV entry or gene delivery to the nucleus. A previous study demonstrated that the *fp25* mutation enhanced infectivity of the BV resulting from better genome integrity and uptake [41]. This could suggest that the entry pathway used by the baculovirus BV is similar in both mammalian and insect cells.

In this study, we investigated whether gene delivery with the HT BacMam vectors could be improved further by pseudotyping BV with a truncated VSV-G protein. Use of VSV-G was based on previous studies that reported enhanced transduction efficiency using baculovirus-mediated viral vectors with *in vitro* and *in vivo* applications [32,42,50,53,54]. The results demonstrated that the VSV-G pseudotyping did improve both the percentage of cells transduced and the EGFP signal intensity in both HK-2 and EndoC-βH3 cells. The absence of detectable EGFP protein by immunoblot in EndoC-βH3 using the non-pseudotyped HT BacMam vector confirms a beneficial role for VSV-G during transduction. These observations are in agreement with previous findings, which indicated that improved cell entry was a major factor for transduction efficiency [32,50]. Pseudotyped baculoviruses expressing either influenza virus neuraminidase, Spodoptera exigua MNPV F protein or human endogenous retrovirus envelope protein have also resulted in better cell entry [50]. Furthermore, increased expression could be enhanced from improved endosomal escape in a VSV-G pseudotyped baculovirus [32].

To gain insight into the feasibility of transducing non-proliferating pancreatic β cells, EndoC-βH3 cells were treated with tamoxifen. Overall, the results showed that the gene delivery and expression of *egfp* was comparable between tamoxifen-treated and non-treated EndoC-βH3 cells when transduced with CMV.EGFP^HT^_VSV-G. This suggests that non-proliferative human beta cells are susceptible to baculovirus transduction and can be targeted for gene therapy using BacMam vectors.

However, the pancreatic islet consists of several different types of cells clustered together and the accessibility of these cells to the BacMam vector will be different to that of monolayer cultures maintained within a laboratory setting. Therefore, to study the BacMam vector as a tool to efficiently deliver genes in pancreatic tissue, we acquired islets that were isolated from cadaveric donor pancreases. The results demonstrated that the BacMam vectors could successfully transduce intact islets from two different donors. Interestingly, the pancreatic islets transduced with CMV.EGFP^HT^_VSV-G showed the highest *egfp* expression compared to all other BacMam vectors tested in this study. These findings are consistent with previous data within this study for transduced HK-2 and EndoC-βH3 cells. To our knowledge, this is the first evidence of *ex vivo* gene delivery in intact human pancreatic islet cells using BacMam, progressing from a previous study characterising *egfp* expression in dispersed islet cells [55].

Although we have demonstrated the promise of a comparatively safe baculovirus-based gene delivery system, further understanding will be required to optimise routes of administration and understand the possible limitations that could arise from translation into clinical application. These challenges include low transduction efficiency, inflammatory response and nonspecific gene delivery along with understanding of the logistical, economic and clinical aspects [50].

## 5. Conclusions

In summary, our findings demonstrate a novel baculovirus-based delivery tool for therapeutic genes that can be used in future, to potentially improve success rates of islet cell transplantation. Using this system, we will further our study to characterise the therapeutic effects of delivering *bcl2*, *sod2* and anti-inflammatory genes into human pancreatic islet cells.

## Figures and Tables

**Table 1:** Quantitative assessment of BacMam transduction of HK-2 cells by flow cytometry.

	24 h Post-Transduction		48 h Post-Transduction		72 h Post-Transduction	
	FB ^1^	HT ^2^	FB ^1^	HT ^2^	FB ^1^	HT ^2^
% transduced HK-2 cells	90	95	99	99	99	99
% transduced HK-2 cells with FITC ^3^ reading above 10^7^	21	42	21	47	10	27

^1^ FB = CMV.EGFP^FB^; ^2^ HT = CMV.EGFP^HT^, ^3^ Fluorescein isothiocyanate.
